# Predicting the academic achievement of students using black hole optimization and Gaussian process regression

**DOI:** 10.1038/s41598-025-86261-y

**Published:** 2025-03-28

**Authors:** Yanyu Chen, Xiaolin Yao

**Affiliations:** 1 School of Education, Durham University, Leazes Road, Durham, DH1 1TA UK; 2 School of Information and Business Management, Dalian Neusoft University of Information, Dalian, 116021 Liaoning China

**Keywords:** Predicting academic achievement, Student performance prediction, Black hole optimization, Gaussian process regression, Psychology, Mathematics and computing

## Abstract

Academic achievement is vital for campus life and education since it indicates the caliber of the teachers, administration, and students’ learning abilities. Issues such as poor study conditions and family disruptions can impede a student’s capacity to achieve. Teachers are looking for practical solutions to these concerns because solving problems one at a time might be tough. This study uses a combination of black hole optimization (BHO) and Gaussian process regression (GPR) algorithms to predict students’ academic success in higher education. The method is divided into three stages: data pre-processing, identification of effective indicators using BHO algorithms, and forecasting of academic performance. The presented approach makes use of the GPR algorithm to choose the relevant features and the weighted combination of GPR models to forecast that the GPR model would be used for the weighting operation that is, to determine the ideal weights. The experimental findings demonstrate that our method has a lower error rate of 0.95 and 0.81 in terms of RMSE and MAE than the competing methods. The proposed method can assist teachers in analyzing student behavioral patterns, understanding academic performance impact mechanisms, and developing effective learning supervision plans.

## Introduction

Academic achievement development is essential to a nation’s progress. High-quality institutions must raise their students’ physical and academic fitness levels in order to develop professional academic achievement and raise the general caliber of their student body^1^. The most important measure of progression in the education system of any nation is student academic performance. Gender, age, the teaching staff, and students’ learning all have an impact on students’ academic achievement. Predicting students’ academic success has drawn great attention in education. To put it another way, student performance is the degree to which students meet both long- and short-term learning objectives^2^. Universities and training facilities are just two examples of the many educational institutions that make up the educational system. Decision-makers in the system of higher education can enhance the quality of instruction in the process of student development by learning how to mine the hidden knowledge contained in these data^3^. The number of college students is rising as society develops and as higher education becomes more widely accepted. The learning and teaching quality is somewhat impacted by teachers’ difficulty in keeping track of each student’s learning circumstances. This causes a certain percentage of college and university students to repeat grades, fail exams, or drop out every year, all of which have a negative impact on the students’ future growth. Higher education has gradually shifted its focus to the caliber of student training. One of the main indicators of how well students are trained in higher education is their academic performance^4^. The ability to forecast and analyze student performance is essential for teachers to identify students’ areas of weakness and support them in raising their grades. Administrators can enhance their operations, just as students can enhance their learning activities^5,6^. The prediction of a student’s future academic success using behavioral data already collected is known as academic achievement prediction. Prediction of academic achievement is one of the first and most widely used uses of educational data mining^7^. In particular, academic performance prediction typically forecasts the trajectory of academic success by gathering student behavioral data and applying statistical or data mining approaches to recognize trends in the data. Academic performance prediction serves the purpose of anticipating the trend in academic performance development ahead of time and identifying potential student issues early on. This lowers the chance of failing the course and enables teachers and students to respond quickly. Research in this area is always evolving, just like the educational environment^8^. In this paper, the combination of the BHO and GPR algorithms is used to predict the academic progress of students at higher education levels. The proposed method consists of three steps, which, after data pre-processing, determine the effective indicators for academic progress using the BHO algorithm. In this process, correlation criteria are used to identify effective indicators. Then, based on the detected indicators, the prediction process is carried out using a weighted ensemble composition based on GPR models, and the prediction is made based on this model. The innovations in the paper are as follows: This study introduces indicators for higher education students’ academic progress, presents a BHO algorithm-based feature selection method, and introduces a GPR-based weighted ensemble system for predicting academic performance. This paper’s key contributions are as follows.

- Introducing a set of indicators relevant to the student’s level of academic progress in higher education.
- Presenting a method for selecting features relying on the BHO algorithm for the problem and determining the most relevant factors to students’ academic progress.
- Providing a GPR-based weighted ensemble system for predicting students’ academic performance.

The paper proceeds as follows: Similar works are examined in Section “Related works”. Then, the proposed approach is detailed in the third section; followed by presenting the results of its implementation in the fourth section. In the fifth section, the research findings are discussed. Finally, “Conclusions” section are reached.

## Related works

Many studies have been done to improve the quality of forecasting students’ academic achievement; in this section, we will cover a few of them.

Zhang et al.^9^ created a class-balance labeled training data set with the goal of learning a sparse graph by examining the features of imbalanced data. They make use of sparse representation and Laplacian score sampling. Despite focusing on processing imbalanced data, many studies neglect to consider the limitations of the data. The efficiency of the classifier for unbalanced data may be impacted by the small amount of data.

Guarn et al.^10^ used cost-sensitive strategies on huge data sets to address the challenges related to imbalanced data caused by a small portion of data sources. Among the many topics that Alturki et al.^11^ addressed was identifying the important traits that can be employed for predicting the performance of the student. The findings showed that the most consistently used characteristics for predicting academic achievement are internal assessments and aggregate evaluation points. Other noteworthy characteristics were also found, such as social traits, past performance, extracurricular activities, and self- and inner assessment. The most often used information-digging techniques were discovered to be decision trees and neural networks.

According to Li et al.^12^, the attendance of a student in a class is the best indicator of academic performance, and academic achievements are somewhat correlated with extracurricular activity participation.

Hadad^13^ discovered that the decisive elements for prediction were academic and family traits. The researchers also found that the most often utilized attributes were the students’ average cumulative grade points and the results of the student’s internal and external exams.

Anoopkumar et al.^14^ demonstrated how to forecast fourth-year undergraduate students’ performance using their pre-university grades and taking into account their scores from their second-year coursework. They disregarded the pupils’ socioeconomic background and family dynamics in favor of utilizing grades alone to predict achievement.

A predictive study methodology was presented by Amrieh et al.^15^ to determine university students’ happiness with an online summer program. A questionnaire with inquiries about demographics, socioeconomic status, and additional factors was distributed to the pupils. In order to understand how learners interacted, that prediction model used regression and ANOVA analysis.

Two prediction models were introduced by Wood et al.^16^ for estimating the performance of students on final exams. The success of the students in their final exams was predicted using an SVM (Support Vector Machine) algorithm and the KNN (K-Nearest Neighbor) method based on their social, class, and demographic characteristics.

To improve the identification of at-risk students, Hung et al.^17^ recommended using a range of classification techniques, including SVMs, random forests, and neural networks. Experiments on two datasets collected from a university and a school context showed that the suggested technique performed better in terms of both sensitivity and accuracy. Using the Pearson correlation, Beckham et al.^18^ investigated potential influences on student performance and found variables that could either help or impede academic progress. Findings reveal that while a mother’s education has a positive effect on academics, prior failures have a detrimental impact on students’ scores. The article also uses machine learning algorithms to predict student grades. With an RMSE of 4.32, the MLP 12-Neuron model performs the best, followed by Random Forest and Decision Tree. By using a feedforward spike neural network, Liu et al.^19^ were able to improve prediction accuracy to 70.8% when predicting student achievements. This technology can positively influence the quality of learning and teaching by assisting college instructors in managing students and creating learning monitoring plans that work.

Moubayed et al.^20,21^ examined the problem of figuring out the level of student participation using the K-means algorithm. The authors also developed a set of rulers that used the Apriori rules for associations approach to relate student engagement to academic success. Analysis of the research data revealed a favorable link between the students’ participation in an online educational atmosphere and their academic success. Using both individual and group methods, Jalota^22^ developed a successful model to forecast academic achievement at the secondary level. The model makes use of three ensemble algorithms (BAG, LogitBoost, and Voting) as well as three single classifiers (MLP, Random Forest, and PART). Classification models are developed in blended versions for improved performance. With a 99.8% accuracy rate, the model beats Logitboost with Random Forest, suggesting its potential for use in learning outcomes in the future. Table 1, includes the summarization of the studied works.

**Table 1:** The reviewed studies.

Reference	Year	Research goal	Method	Limitation
Guarn et al.^10^	2015	Predict low academic performance	Cost-sensitive strategies on large datasets	Limited to specific enrollment context.
Alturki et al.^11^	2021	Predict student academic performance	Data mining techniques (decision trees, neural networks)	Does not consider the impact of external factors like socioeconomic status.
Li et al.^12^	2019	Identify factors impacting student performance	Correlation analysis	Limited to specific factors (attendance, extracurricular activities).
Hadad^13^	2017	Identify factors impacting student performance	Not explicitly mentioned	Limited to academic and family factors.
Anoopkumar et al.^14^	2018	Predict fourth-year undergraduate performance	J48 classification	Neglects socio-economic and family factors.
Amrieh et al.^15^	2016	Predict student satisfaction with online summer program	Regression and ANOVA analysis	Limited to specific program context.
Wood et al.^16^	2017	Predict student performance on final exams	SVM and KNN	Limited to specific demographic and social factors.
Hung et al.^17^	2019	Identify at-risk students	SVM, random forests, neural networks	Limited to university and school contexts.
Beckham et al.^18^	2023	Identify factors affecting student performance	Pearson correlation and machine learning	Limited to specific factors and machine learning models.
Liu et al.^19^	2022	Predict student academic performance	Feedforward spike neural network	Limited to specific context and model.
Moubayed et al.^20,21^	2020, 2018	Relate student engagement to academic success	K-means clustering and association rule mining	Limited to specific e-learning environments.
Jalota^22^	2023	Predict academic performance	Ensemble and single classifiers	Limited to specific context and model selection.

## Research methodology

This section first mentions the method for collecting the necessary data to conduct the research, as well as the specifications of these data. It subsequently presents the introduced approach for the prediction of the student’s academic performance in higher education.

### Data

Data for this study were gathered by the distribution of questionnaires to Nanjing, China, university students. This dataset comprises information on 628 students from two technical and engineering faculties. During the questionnaire distribution process, the student’s academic and educational conditions were documented at the start of the educational period. Subsequently, the average grades of the respondent students were compiled as the target variable after the academic term. Within this dataset, 383 records pertain to female students, while the remaining samples correspond to male students. The age range of the participants is 20–31 years, with an average age of 23.78 years. Table 2 contains a list of the information available in the database.

**Table 2:** Database specifications.

ID	Title	Type
I1	Place of Study	Nominal Discrete
I2	Gender	Nominal Discrete
I3	Age	Numerical Continuous
I4	Residential Status	Nominal Discrete
I5	Number of Members in Family	Numerical Discrete
I6	Parents’ Marital Status (Divorce Status)	Nominal Discrete
I7	Mother’s Education Level	Nominal Ordinal
I8	Father’s Education Level	Nominal Ordinal
I9	Employment Status of Mother	Nominal Discrete
I10	Employment Status of Father	Nominal Discrete
I11	Justification for Study Location Selection	Nominal Discrete
I12	Student’s Legal Guardian	Nominal Discrete
I13	Time Distance Between Residence and Place of Study	Numerical Continuous
I14	Weekly Study Duration	Numerical Continuous
I15	Average Grades Last Term	Numerical Continuous
I16	Having a Scholarship	Nominal Discrete
I17	Parents’ Financial Support for Education	Nominal Discrete
I18	Use of Extra Classes	Nominal Discrete
I19	Having Extracurricular Activities	Nominal Discrete
I20	History of Participation in Academic Competitions	Nominal Discrete
I21	Desire to Continue Education	Nominal Discrete
I22	Internet Access at the Residence	Nominal Discrete
I23	Emotional Relationship Status	Nominal Discrete
I24	Degree of Relationship Quality with Family Members	Nominal Ordinal
I25	How Much Free Time There is After Classes	Numerical Continuous
I26	Communication with Classmates Outside of Place of Study	Nominal Discrete
I27	Consuming Alcohol Throughout the Week	Nominal Discrete
I28	Consuming Alcohol Throughout Weekend Holidays	Nominal Discrete
I29	Current Health Situation	Nominal Ordinal
I30	Total Absences in the Class	Numerical Continuous
-	Student’s Final Average Grades	Numerical Continuous

Referring to Table 2, the gathered dataset encompasses 30 independent features related to students’ academic information and lifestyle, with the dependent variable indicating the students’ average final grades, reflecting their academic accomplishments. For instance, age is represented as a natural number of years. Features such as ‘Time Distance Between Residence and Place of Study,’ ‘weekly study duration,’ and ‘amount of free time after classes’ are quantified in minutes. The residence status indicates whether the student resides in a dormitory, an independent house, or with parents. Additionally, the parents’ educational level can be categorized as parents’ educational level can be categorized as 1- Illiterate, 2- High school diploma, 3- Bachelor’s degree, 4- Master’s degree, and 5- Doctorate or higher. The parent’s employment status may fall into one of the following classes: 1- Education, 2- Healthcare, 3- Services, 4- Legal, 5- Government, and 6- Other. The reason for selecting the educational institution is categorized into several scenarios: 1- Personal interest, 2- Proximity to residence, 3- Cost, and 4- Other reasons. Additionally, the legal guardian may be categorized as 1- Parents or 2- Other. These features are represented either numerically or through a rating system. Furthermore, other nominal features of the database are defined as logical features with values of true or false. The target parameter in this dataset is the students’ average final grades, represented as a numerical value ranging from 0 to 20.

### Proposed method

**Fig. 1:**
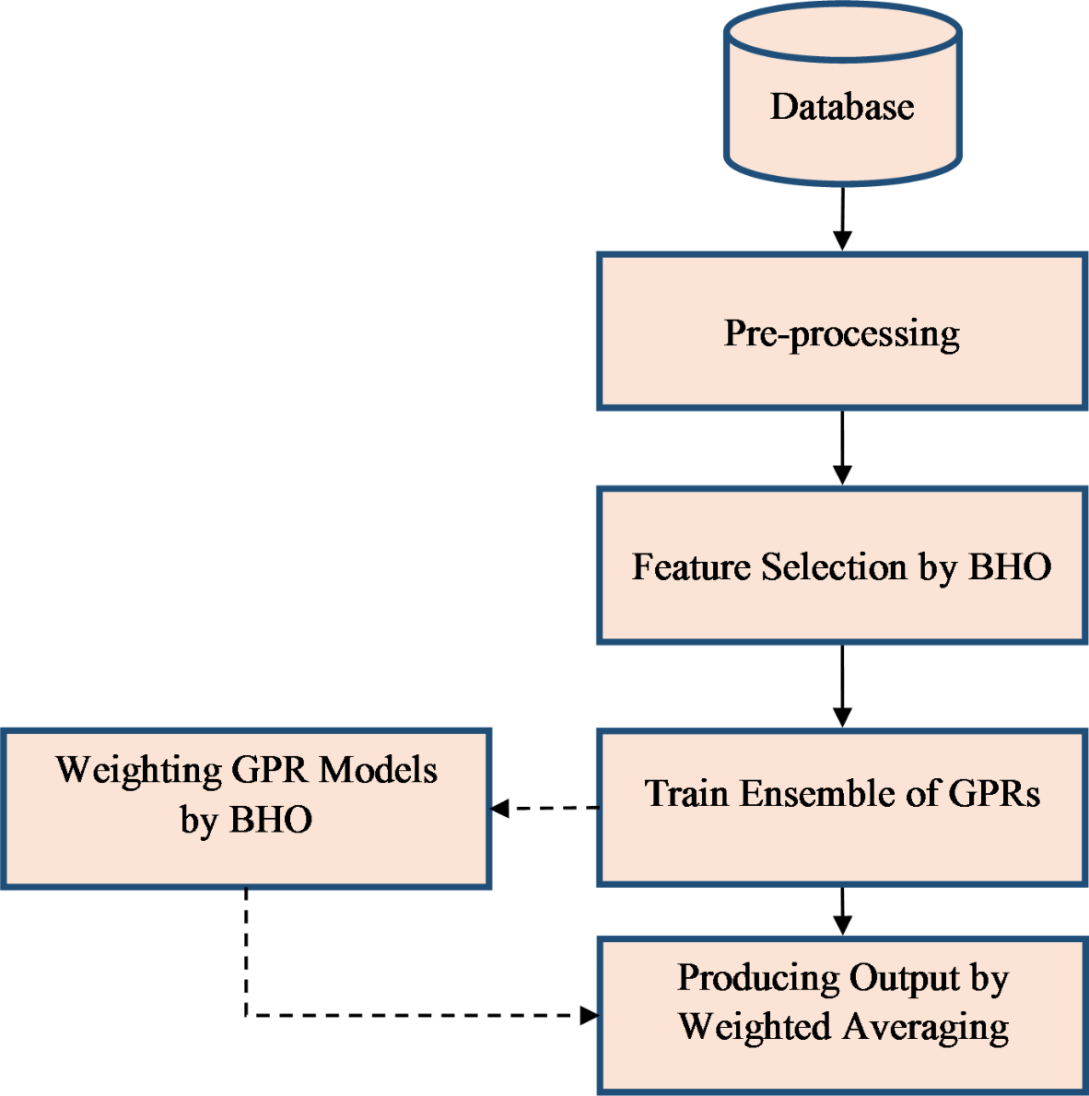
Diagram of the steps of the proposed method.

Utilizing a dataset relevant to the objective, along with employing a robust learning model, are fundamental prerequisites for developing an efficient system to predict students’ academic progression. This study employs a feature selection approach based on BHO to fulfill the first prerequisite, while simultaneously striving to satisfy the second prerequisite by utilizing an ensemble system predicated on a weighted combination of GPR models. The proposed system in this study predicts the academic progress of students in higher education through three principal steps (refer to Fig. 1).

1. Data preprocessing.
2. Identification of impactful indicators on academic progress by BHO.
3. Prediction of academic performance based on a weighted combination of GPR models.

The gathered data are initially pre-processed utilizing a straightforward mechanism in the suggested manner. The next step is to determine which indicators are most important for tracking students’ academic development using a BHO-based technique. The goal of this feature selection method is to choose indicators with the least amount of redundant data and the maximum degree of relevance to the target variable. Following the identification of the variables that affect students’ academic development, an ensemble system based on a weighted mixture of Gaussian Process Regression (GPR) models is trained using the chosen set of data. Several GPR models in this ensemble system are trained on different groups of data. These models are then ranked according to the caliber of their training with BHO. During this stage, the optimization method aims to determine the weight values for every GPR model so that the ensemble system’s weighted average of their outputs produces the least amount of prediction error.

#### Preprocessing

The purpose of data pre-processing is to prepare the data for the estimation of students’ academic progress by machine learning algorithms. In this section, after managing records with missing values and converting nominal values to numerical, a dataset comprising entirely of numerical values is obtained. The suggested technique then makes use of this dataset in the estimate procedure. The suggested approach imputes the average of the current values for a numerical attribute that is missing from a record in order to manage records with missing values. Furthermore, the label that appears the most frequently for a given property is used to replace missing values for nominal attributes. All nominal properties are transformed to numerical form following the pre-processing stage. For example, if an attribute consists of the values {‘yes’, ‘no’}, the attribute’s values are converted to the corresponding numerical set {1, 0}.

#### Feature selection based on BHO

The BHO method is used to identify notable characteristics from a group of candidate factors. BHO is an algorithm relying on improving the population with characteristics like a simple mechanism and an efficient search of the issue space, allowing it to be employed for solving a wide range of problems. Each potential solution is depicted as a star in this method, while the best answer is considered as a black hole. The system replicates stellar motion patterns as well as black hole behavior while devouring nearby stars. By using this technique, potential solutions are kept out of local optima^23^. Because of these abilities, BHO is regarded as an effective approach for addressing the problem of selecting features in this study. To do so, the challenge of picking significant characteristics must be expressed as an optimization problem. Each possible indication is modeled as an optimization variable in the proposed technique. As a result, the BHO algorithm’s optimization variables correspond to the amount of potential factors. Every variable may have a binary value of one, which indicates the selection of a feature, or zero, which indicates the elimination of the feature. For the BHO approach, this means that every solution vector is encoded as a binary string of length thirty. Every indicator vector could offer a viable way to find pertinent indicators for predicting students’ academic success. The suggested optimization approach selects indicators relying on the criteria of correlation. The goal is to select as few variables as possible that have the highest link with students’ academic success:

1 $$\:Objectiv{e}_{1}\left(\overrightarrow{x}\right)=\frac{1}{K}\sum\:_{k=1}^{K}\left|Corr\left({\overrightarrow{x}}_{k}.Q\right)\right|$$

where K, which can be found by counting the total amount of “1” values in the solution, stands for the number of chosen indicators. The k-th chosen factor in the solution (x) is represented by the variable (x_k). The function (Corr(X, Y)) calculates the correlation among two indicator vectors, (X) and (Y). Finally, (Q) represents the target vector of students’ academic performance. Actually, the average correlation among the chosen factors and the target variable is determined by Eq. (1).

Conversely, there shouldn’t be any redundant data in the set of chosen indicators. In order to meet this requirement, the correlation between the chosen indicators is calculated, and an attempt is made to reduce this measurement. The following formulation is used for this objective:

2 $$\:Objectiv{e}_{2}\left(\overrightarrow{x}\right)=\frac{1}{{K}^{2}}\sum\:_{i=1}^{K}\sum\:_{j=1}^{K}\left|Corr\left({\overrightarrow{x}}_{i}.{\overrightarrow{x}}_{j}\right)\right|$$

The mean correlation among each pair of selected indicators is ascertained using the previously given equation. The suggested strategy is to reduce this goal. The goals of goal 1 (which needs to be maximized) and goal 2 (which needs to be minimized) can be combined in the following way to determine the fit of each solution in the suggested method:

3 $$\:Fitness\left(\overrightarrow{x}\right)=\frac{\frac{1}{{K}^{2}}\sum\:_{i=1}^{K}\sum\:_{j=1}^{K}\left|Corr\left({\overrightarrow{x}}_{i},{\overrightarrow{x}}_{j}\right)\right|}{\sqrt{K}(1+\frac{1}{K}\sum\:_{k=1}^{K}\left|Corr\left({\overrightarrow{x}}_{k},Q\right)\right|)}$$

In the preceding equation, the numerator seeks to reduce the correlation between the selected indicators, while the denominator assesses the correlation between the selected indicators and the goal variable. In addition to the previously mentioned objectives, the proposed feature selection method also seeks to limit the total amount of selected indicators. This objective is achieved by incorporating the coefficient ($\:\sqrt{k}$) in the equation. The BHO algorithm uses the subsequent processes to choose indicators that are pertinent to students’ performance:

Step 1: First, create a random starting population of binary strings that represent the solutions.

Step 2: Using Eq. (3), determine each solution’s fitness.

Step 3: Select the solution to be described as the black hole ($\:{X}_{BH}$) that has the lowest fitness.

Step 4: Adjust each solution’s position, indicated by ($\:{X}_{i}$), as stated in^23^:

4 $$\:{X}_{i}={X}_{i}+rand.({X}_{BH}-{X}_{i})$$

In this case, the star i’s position throughout the problem space is represented by the vector ($\:{X}_{i}$), and the black hole’s position is indicated by the vector ($\:{X}_{BH}$). Furthermore, “$\:rand$” is a random value in the interval of (0,1).

Step 5: Determine the cutoff distance^23^ for the star that the black hole will ingest^23^:

5 $$\:R=\frac{fitness\left(BH\right)}{\sum\:_{i=1}^{N}fitness\left(i\right)}$$

where N denotes the population size in BHO.

Step 6: Substitute the black hole with another solution if its fitness is greater than the existing black hole’s.

Step 7: Substitute new, random solutions for any existing ones that are nearer to the black hole than the minimum threshold (R).

Step 8: Go to the subsequent step if the algorithm’s iteration counter has surpassed the limit T; if not, start the search over from Step 2.

Step 9: As the best answer, deliver the black hole having the smallest fitness value.

The chosen indications are taken out of the optimal solution that was found using the previous phases, and in the following step, they are used as input for the suggested learning model.

#### Prediction based on GPR

In the final step of the presented approach, GPR-based learning models are utilized to predict students’ academic progress using selected pertinent indicators. The proposed model employs a weighted ensemble of GPR models to achieve this objective. This technique can contribute to the development of a generalizable and more comprehensive learning model that addresses the limitations of each learning model. This mechanism encompasses two more detailed steps: ‘training GPR models and forming an ensemble model,’ followed by ‘ranking the GPR models.’ Each of these steps is elaborated upon in the subsequent section.

##### Construction of an ensemble model based on GPR

In the proposed model, a fusion of multiple GPR learning models is employed within an ensemble system to forge a more robust model for predicting students’ academic progress. In this model, the training dataset is partitioned into K = 10 subsets with overlapping members, and subsequently, K learning models are trained on these subsets. Each GPR model is constructed using one of the training datasets, and ultimately, the prediction of academic progress in new samples is achieved by averaging the outputs of these models. Theoretically, it has been proven that ensemble learning can be effective in improving the performance of machine learning models^24^.

From a machine learning perspective, a Gaussian process (GP) involves lazy learning and the measurement of similarity between data points (using the kernel function) to predict new points from the training data. GPs can be regarded as an infinite-dimensional multivariate Gaussian distribution^25^. They are beneficial for statistical modeling because they leverage the intrinsic properties of the normal distribution. On the other hand, it is effective for predicting dependent variables using independent variables that follow a normal distribution^26^. A continuous random process such as $\:\{{X}_{t};t\in\:T\}$ is Gaussian if and only if, for every finite set of indices $\:{t}_{1},\dots\:,{t}_{k}$ in the set T, the joint distribution of $\:{X}_{{t}_{1},\dots\:,{t}_{k}}=({X}_{{t}_{1}},\dots\:,{X}_{{t}_{k}})$ is multivariate normal. Consequently, every multivariate combination of $\:({X}_{{t}_{1}},\dots\:,{X}_{{t}_{k}})$ has a Gaussian (or normal) distribution.

Regression may be performed in a non-parametric, Bayesian manner with the help of the GPR, which makes machine learning applications easier to use. Among its many benefits are its capacity to produce confidence intervals for the estimated values in forecasts and its efficient performance on tiny datasets. Unlike many well-known supervised machine learning methods that learn exact values for every parameter of a function, the Bayesian approach concludes distributions of probability over all possible values. GPR is not restricted to a particular functional form since it is non-parametric^26^. Rather than determining the probability distribution of variables for a specific function, GPR computes the probability distribution over all functions that correspond to the data. With this method, a starting prior probability (in the function space) is determined, and the projected distribution at the designated locations is obtained by computing posterior probabilities with the training data. Using an average function and covariance function, a default GP may be defined in the initial step of the GPR process. Specifically, a GP is akin to an unbounded multivariate Gaussian distribution, in which each set of data labels has a common Gaussian distribution. In this default GP, prior knowledge about the function space can be combined by selecting appropriate mean and covariance functions.

##### Ranking of GPR models in a BHO-based ensemble system

BHO is used to rate the trained learning models following the training of the GPR models. Finding the relevance coefficient of each algorithm’s output in the suggested method’s final output is the aim of this ranking. As such, in this BHO example, the optimization variables match the collection of ideal coefficients for the estimating models used in the previous stage. In other words, each BHO solution vector is ten-dimensional, with each dimension indicating the significance coefficient of the outputs from GPR1 to GPR10 in computing the output of the proposed method. The search bounds for the optimization variables are considered as a real value in the interval [0, 1]. Given the detailed computational steps of the BHO algorithm in Sect. 2.3.2, this section focuses solely on the fitness function employed for BHO. The fitness function employed in BHO to rank the outputs of the learning model based on the Root Mean Squared Error (RMSE) criterion is described as follows:

6 $$\:fitness=\sqrt{\frac{1}{n}\:\sum\:_{i=1}^{n}{({O}_{i}-\frac{\sum\:_{j=1}^{C}{w}_{j}\times\:{Y}_{i}^{j}}{\sum\:_{j=1}^{C}{w}_{j}}\:)}^{2}}$$

In Eq. (6), $\:{O}_{i}$ is the ground-truth value for sample i. Additionally, $\:{Y}_{i}^{j}$ denotes the outputs estimated by the GPR_j_ model for training instance i, and $\:{w}_{j}$ shows the rank given to the GPR_j_ model. Ultimately, n denotes the number of training samples utilized for the fitness evaluation, and C represents the count of GPR models.

This algorithm assigns a coefficient to each GPR model within the ensemble system, which indicates its importance in determining the final predicted values of the ensemble system. It is significant to keep in mind that GPR model ranking operates just once, following the training phase. The output value is first generated independently for each estimating algorithm in the testing process of the suggested approach. The ultimate result is then ascertained through combining these values in accordance with the calculated ranks. In this case, the following formula is used to get the final output of the suggested procedure for each test instance:

7 $$\:{F}_{i}=\frac{\sum\:_{j=1}^{C}{w}_{j}\times\:{Y}_{i}^{j}}{\sum\:_{j=1}^{C}{w}_{j}}$$

## Research finding

The proposed approach is implemented using MATLAB 2020a software. Apart from utilizing the cross-validation methodology with ten iterations in the experimental setup, we also evaluated the proposed method according to the predetermined standards.

### Evaluation metrics

After completing the prediction using the recommended method, we compared the actual and expected values and evaluated the model’s efficacy using measures of root mean square error (RMSE), mean absolute error (MAE), and the concordance correlation coefficient (CCC). RMSE and MAE, calculate the discrepancies between values that an estimate or model predicts and actual values. The square root of the means of the squared differences between the expected and actual values is used to compute the RMSE. It is frequently employed to assess a prediction model’s prediction efficiency. One way to express root mean square error is^27^:

8 $$\:RMSE=\sqrt{\frac{1}{N}{\sum\:}_{i=1}^{N}{\left({y}_{i}-{\widehat{y}}_{l}\right)}^{2}}$$

The mean absolute error between the values that were predicted on the test set and the actual values is known as the MAE. There exists a negative correlation between the MAE and the prediction effect. The real prediction error can be precisely represented by this statistic. Consequently, it was used to assess the inaccuracies of various prediction models. One method for calculating the MAE is^27^:

9 $$\:MAE=\frac{1}{n\:}\left|{y}_{i}-{\stackrel{-}{y}}_{i}\right|$$

The CCC measures how well two continuous variables agree with one another. It is especially helpful in determining the degree of agreement between two quantitative measures since it assesses both precision and accuracy. The following equation is used to determine CCC^28^:

10 $$\:CCC=\frac{2{\sum\:}_{i=1}^{d-1}\sum\:_{j=i+1}^{d}{\sigma\:}_{ij}}{\left(d-1\right){\sum\:}_{i=1}^{d}{\sigma\:}_{i}^{2}+{\sum\:}_{i=1}^{d-1}{\sum\:}_{j=i+1}^{d}({\mu\:}_{i-}{\mu\:}_{j}{)}^{2}}$$

### Comparative analysis

We assessed the suggested approach in several modes to determine the efficacy of each technique employed in this method in order to demonstrate the method and its methods’ efficacy. Proposed method: This paper uses a feature selection approach based on BHO and an ensemble system based on GPR models to predict academic progress in higher education students through three principal steps: data preprocessing, identifying impactful indicators, and predicting performance.

Proposed (no FS): The feature selection operation is ignored and the prediction operation is performed based on all the indicators listed in Table 2.

Conventional Ensemble: This is related to the case where the combination of GPR models is used for prediction, but instead of using their weighted combination, the averaging operator is used to determine the output of the ensemble model.

Single GPR: It is related to the situation where the academic performance of students is predicted based on the selected characteristics and is done only by using a GPR model.

We also compared the proposed method with two papers of references^18] and [19^.

### Experimental results

We completed the tests based on the circumstances and criteria outlined in the preceding parts, and the pertinent results are presented in this section.

**Fig. 2:**
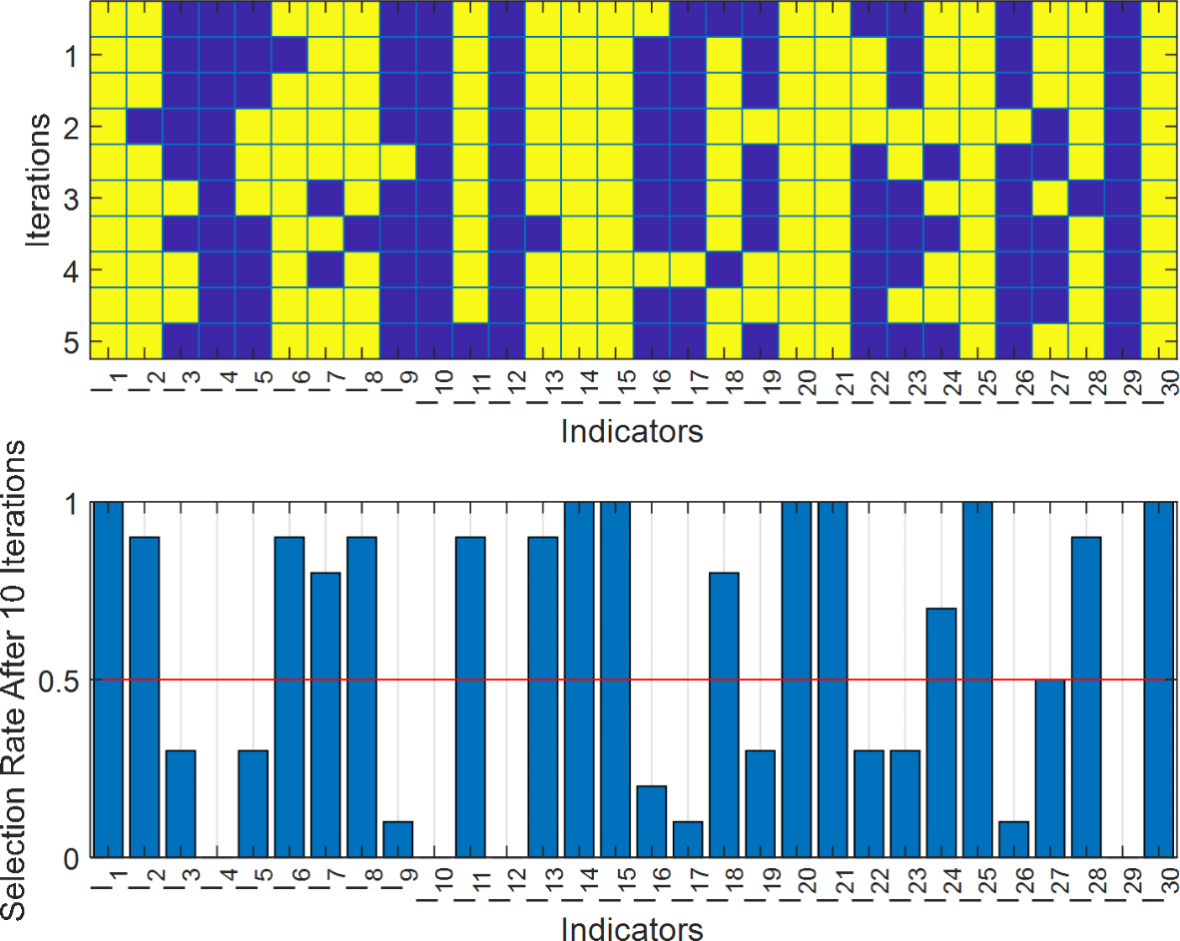
Algorithm for feature selection in the academic achievement of students.

Figure 2 shows the algorithm for feature selection. This diagram has two parts. The upper part shows how features are selected in different iterations. In this section, there are 30 indicators on the horizontal axis and 10 repetitions on the vertical axis. Yellow dots indicate features that have been selected. For example, feature I1 is selected in all 10 iterations and feature I2 is matched in 9 iterations, while feature I4 is not selected in any of the iterations. In the lower part of the graph, the selection rate of each feature in different iterations is displayed. The features that are selected more than 50% in the iterations can be considered as relevant features that are distinguished from other features.

Figure 2 demonstrated that 17 out of 30 candidate features are relevant to the student performance. These features include parents’ divorce status and their education level, conditions of place of study, student alcohol consumption, and student behavioral features such as duration of study, participation in classrooms and other related activities, and his/her previous grades. These features had the most correlation with the performance of the student and at the same time, provided new information which was implied from their low correlation with other features. It should be noted that several features had a high correlation with the target variable, but because of providing repetitive/redundant information, they were discarded by BHO during feature selection. For example, the employment status of the parent, despite having a high correlation with the student performance, was highly correlated with their education level. Also, the student’s legal guardian, financial support, and quality of relationship with family members were highly correlated with the divorce and residential status. Also, the features I25 and I26 were highly correlated with the features I14 and I13, respectively.

**Fig. 3:**
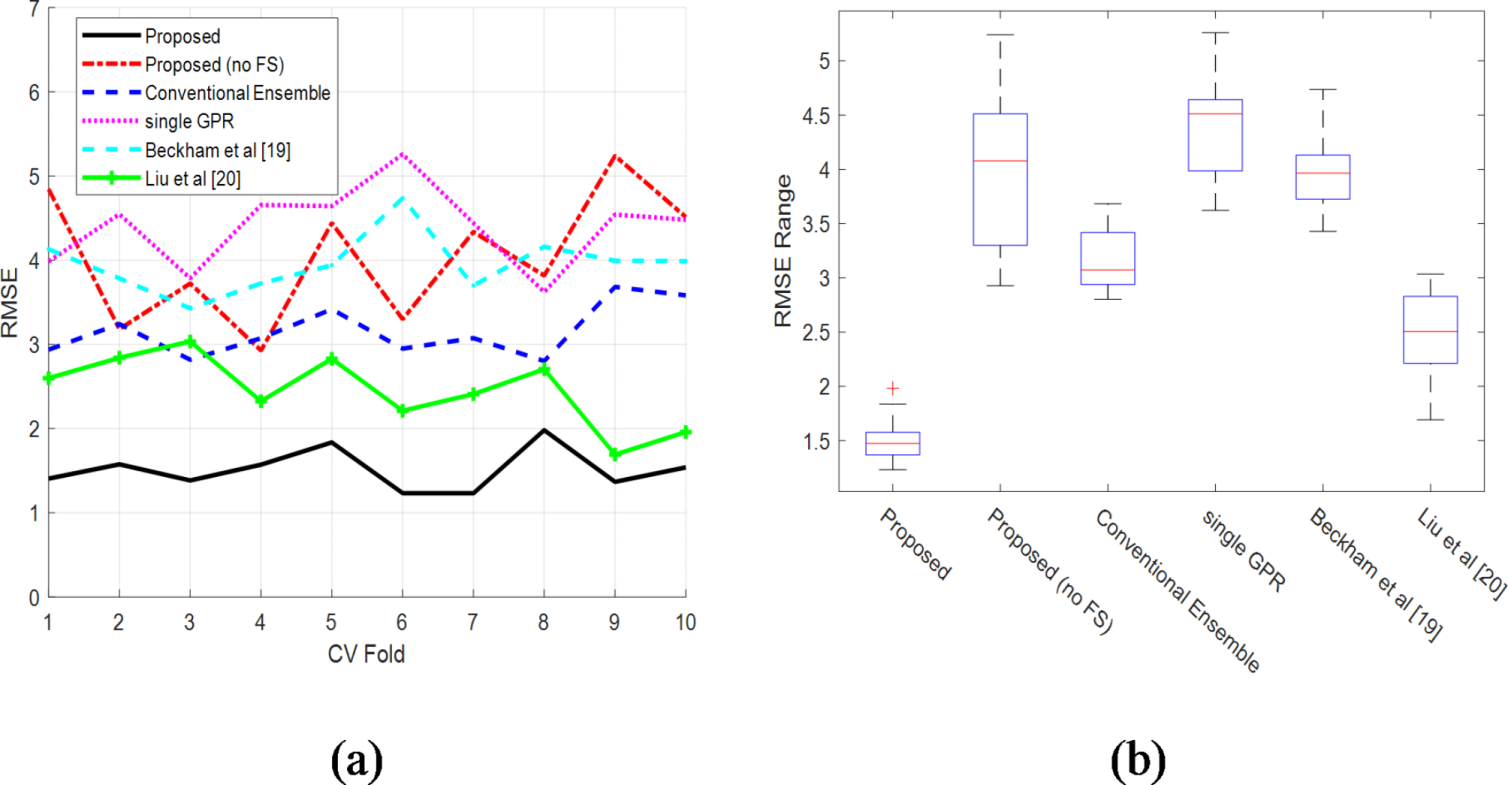
The amount of RMSE in the academic achievement of students: (**a**) RMSE values in each repetition (**b**) Box plot of RMSE.

Figure 3 shows the performance of different methods based on RMSE. Figure 3a shows the amount of RMSE in different iterations of the algorithm. This graph shows that in all iterations, the RMSE of the proposed algorithm in students’ academic performance was lower than other methods. Also, the closest method^19^ also has a significant difference interval compared to the proposed method, which shows this difference is equal to 3.40. Figure 3b shows the intervals of error changes in the form of boxplot diagrams. Each method’s performance is split into four sections on this graph, and each method’s RMSE error is shown in four distinct quadrants. The middle of the box indicates the median RMSE value, while the top and lower lines of each quartile show the blue end limits of that quartile. This diagram shows two important things. First, our proposed method has a lower value of RMSE than other methods in different iterations. Second, the amount of RMSE changes in the proposed method is more limited than other methods in different intervals, especially in tighter intervals, which indicates the higher reliability of the proposed method than the compared methods.

**Fig. 4:**
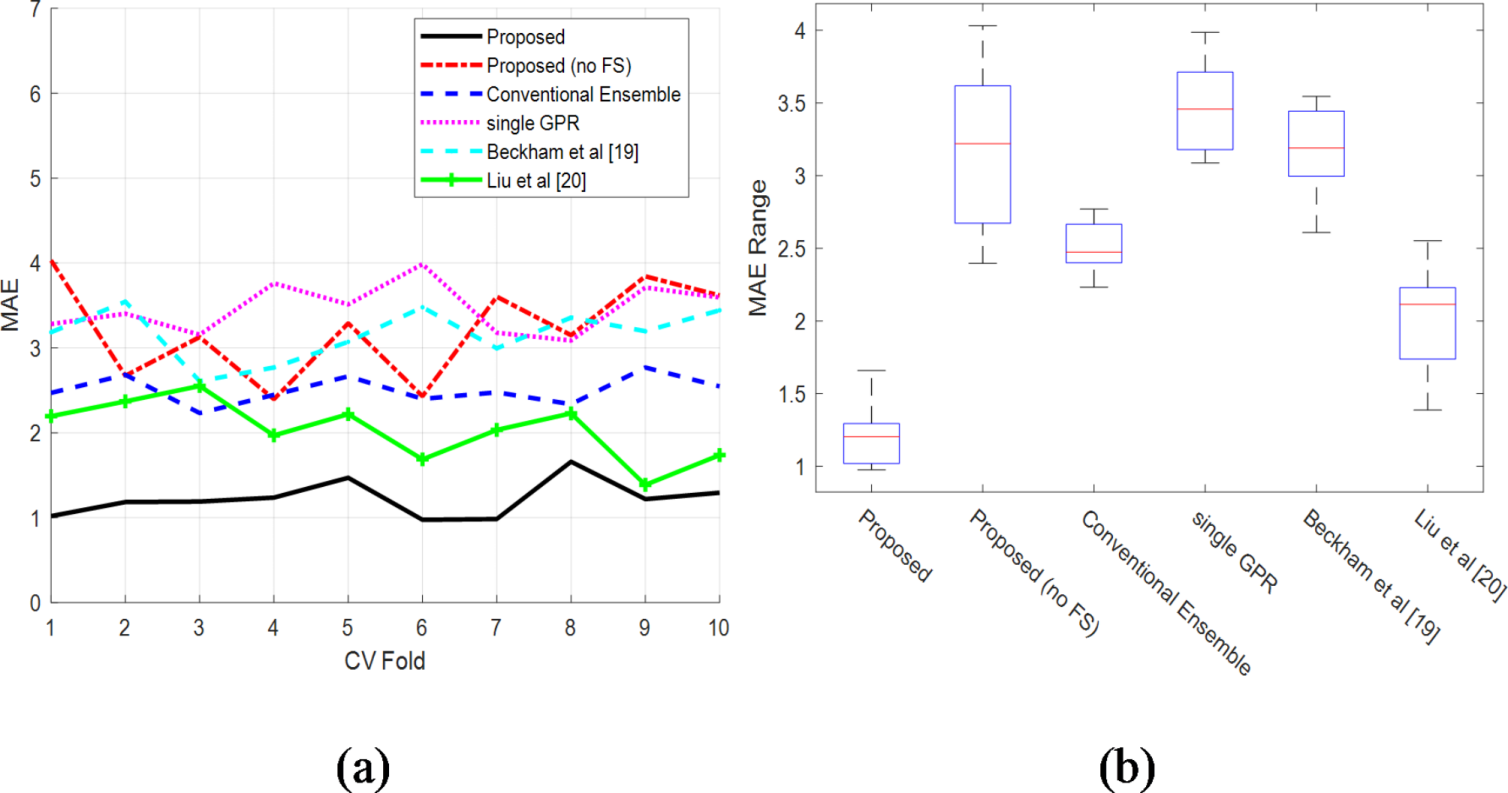
The amount of MAE in the academic achievement of students: (**a**) MAE values in each repetition (**b**) Box plot of MAE.

The performance of several methods based on MAE is displayed in Fig. 4. The quantity of MAE in different algorithm iterations is shown in Fig. 4a. This graph shows that when compared to alternative approaches, the MAE of the suggested algorithm was lower throughout all iterations in terms of students’ academic progress. Furthermore, the nearest technique^19^ has a substantial difference interval when compared to the suggested method, with this difference equal to 0.81. Figure 4b depicts the error change intervals as boxplot graphs. The performance of each method is separated into four portions in this graphic, and the MAE error rate of each approach is displayed in four different quadrants. The upper and lower lines of each quartile show the quartile’s end limits in blue, and the middle of the box depicts the MAE median value.

**Fig. 5:**
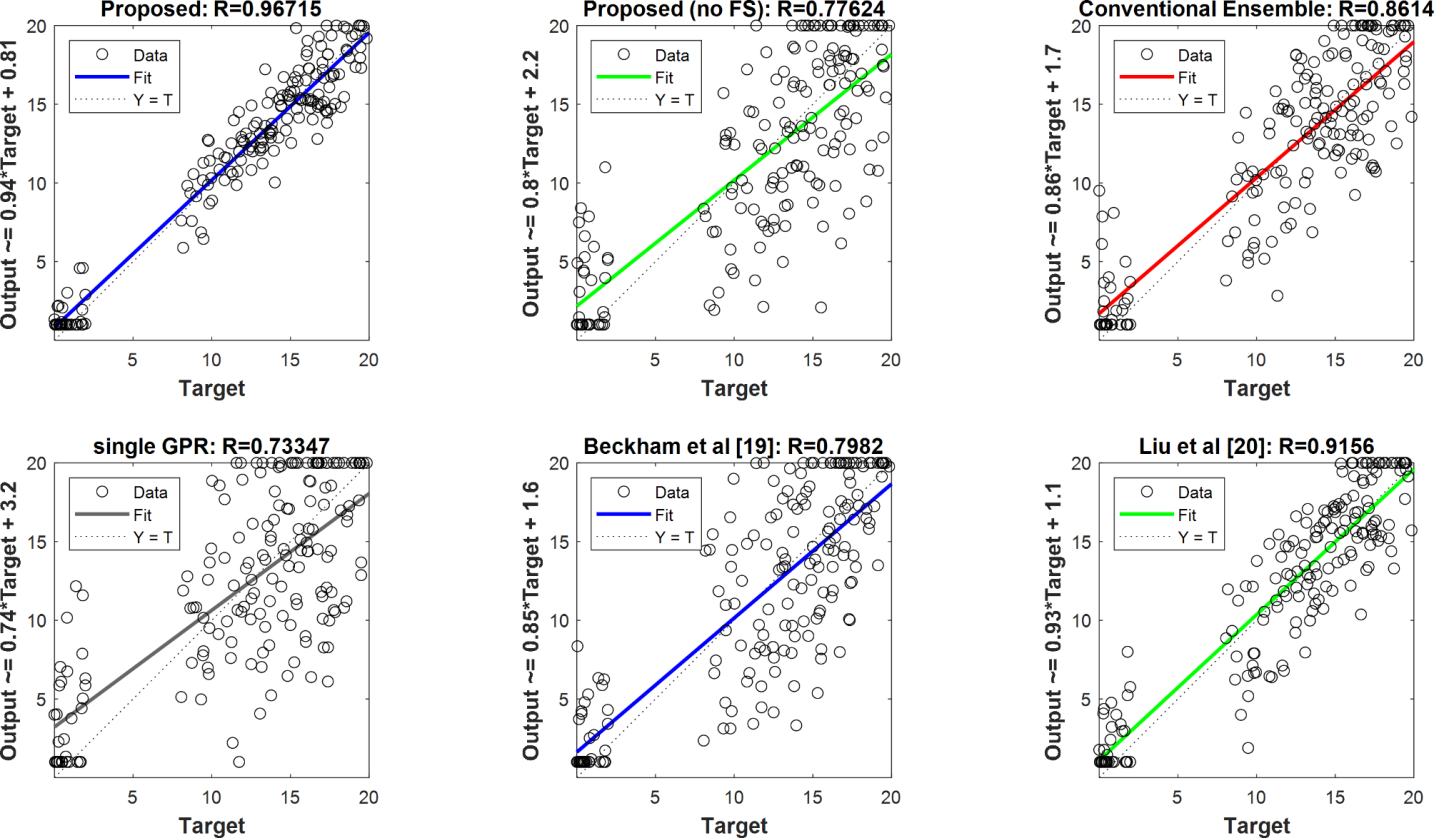
Linear regression chart for the academic achievement of students.

The linear regression diagram is displayed in Fig. 5. The correlation between the actual values and the values predicted by the suggested technique is displayed in the regression figure. These regression charts make it evident that the results predicted by the suggested approach match the actual results more closely. The correlation between the output and the actual value is indicated by the value of R, which is displayed above each graph. An increase in R when compared to the other techniques suggests that the suggested method is more successful in predicting students’ academic development. These graphs demonstrate that our technique is able to produce predictions that better match the actual values and are more consistent than the methods that were compared.

**Fig. 6:**
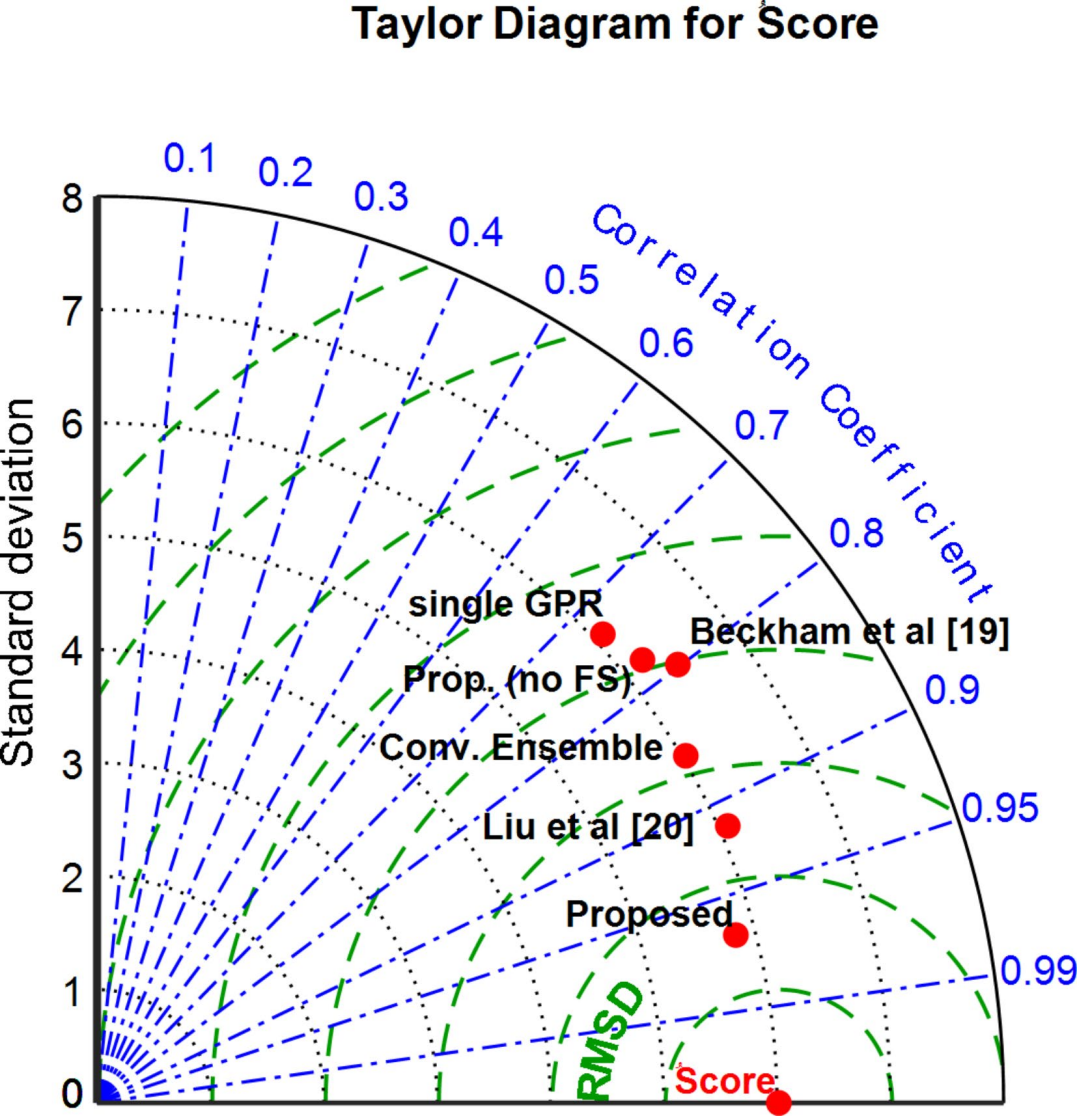
Taylor diagram displaying the effectiveness of the evaluated proposed method.

This is the Taylor diagram in Fig. 6. Based on the Taylor diagram, it is evident that the performance of each approach increased with decreasing distance from the reference point. The suggested approach has a lesser quantity of RMSE and a stronger correlation, as the graph demonstrates. Furthermore, compared to other approaches, the suggested method’s standard deviation is smaller. In other words, the performance of the proposed method has been better than the comparative method in three aspects, i.e. standard deviation, RMSE, and correlation. This superiority can depend on two factors; the first factor is the use of the feature selection method based on BHO, which has been able to remove irrelevant features, and the second factor is the use of the weighted combination of GPR models; because this combination has shown better performance than simple GPR models and simple ensemble systems.

**Fig. 7:**
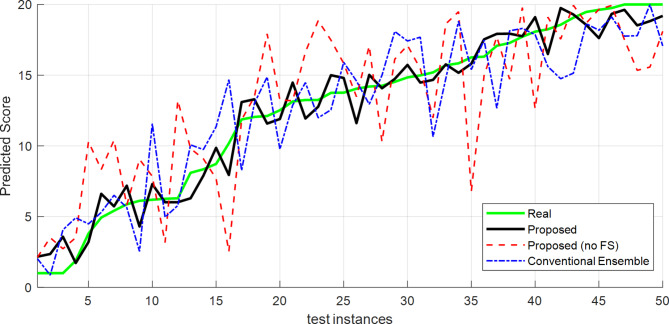
Real values and model-predicted values.

An example of the predictions made by the proposed method is shown in Fig. 7 for its different modes. The green line represents the actual performance of the students, and the other lines represent different states. From this graph, it is clear that the proposed method has outputs that are more consistent with the actual performance of students, and their difference with different modes is less. It should be noted that these outputs are displayed based on 50 samples from the database.

**Fig. 8:**
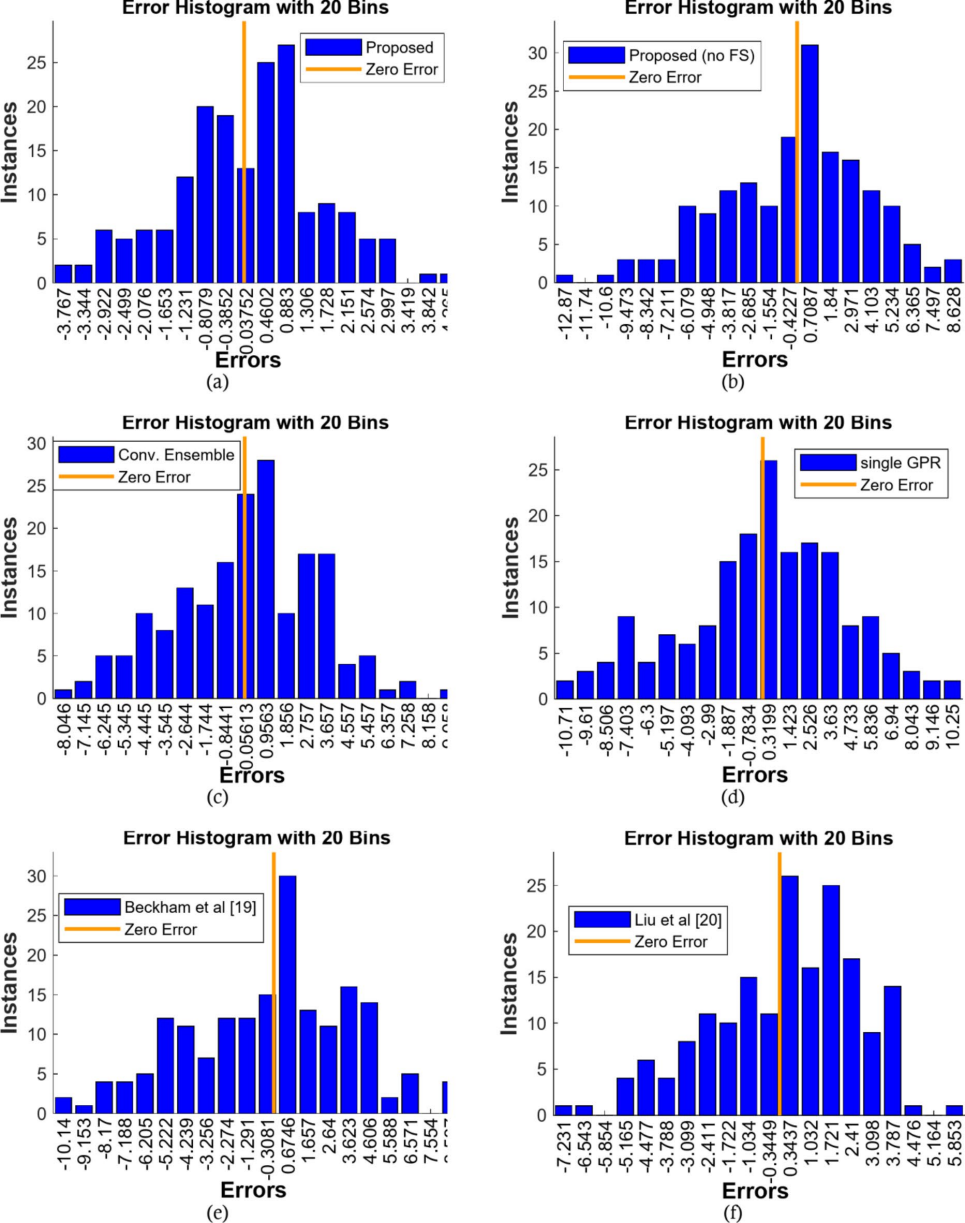
Evaluation of error histograms.

The error histogram is depicted in Fig. 8. The maximum range of error changes for the proposed method is -3.76 to 4.26, which is a larger range than the comparable techniques, indicating that the proposed method’s error rate has a smaller range, indicating that the outputs provided by the proposed method can be used with more confidence.

As seen by the comparison in Table 3, the suggested technique outperformed other comparable methods in addition to performing much better for the RMSE, MAE, and CCC criteria. These findings demonstrate that the suggested approach may be applied as a very dependable and successful modeling and forecasting technique.

**Table 3:** The efficiency of the proposed model and more comparison approaches.

Methods	RMSE	MAE	CCC
Proposed	1.5127	1.2234	0.9666
Proposed (no FS)	4.0326	3.2162	0.7753
Conventional Ensemble	3.1581	2.5030	0.8614
Single GPR	4.3959	3.4672	0.7333
Beckham et al.^19^	3.9588	3.1651	0.7962
Liu et al.^20^	2.4607	2.0381	0.9151

## Discussion, implications, and limitations

This section discusses the empirical evaluation of the proposed method along with its application and possible drawbacks. Through exploring these aspects, we intend to give a clear explanation of the findings and their implications.

### Performance analysis

The combined BHO and GPR method outperformed the prior methods in the prediction of student academic achievement. From Table 3, it is clear that the proposed method has produced the lowest RMSE and MAE and the highest CCC, which ensured better prediction accuracy, precision, and conformity to the actual values.

When the BHO was used for feature selection, the error was reduced significantly. The improvement obtained by the proposed method was a 62.5% decrease in RMSE and a 62.1% decrease in MAE in comparison with the “Proposed (no FS)” method. This proves the effectiveness of determining relevant features in reducing the prediction error which was achieved by the proposed feature selection mechanism.

On the other hand, the proposed weighting strategy in the ensemble GPR model also made a positive contribution to performance enhancement. The proposed method was 52.1% better in terms of RMSE and 47.2% better in terms of MAE than the “Conventional Ensemble” method. These results, support our claim about the effectiveness of determining weights of learning components of the ensemble system dynamically to improve its reliability.

Further, the proposed method yielded a lower RMSE by 61.4% compared to the methods reported in^18^ and by 38.5% compared to^19^. Likewise, it had a 59.3% and 40.0% decrease in MAE than these prior researches were able to attain. As these numerical results show, the proposed method offers significantly better accuracy of predictions and lower errors than the existing methods. These superior results were mainly due to the use of BHO for feature selection and the weighted ensemble GPR model.

### Practical implications

Based on the research results, there are several implications that may be valuable for educational organizations and policymakers. The proposed method may be used as a helpful instrument for early detection of academically at-risk students and subsequent interventions. With the help of accurate prediction of students’ performance, instructors will be able to identify those students who need help and support in their studying process.

In addition, it is established that the features that have a significant influence on the student’s academic performance can guide educational policies and practices. For example, a student’s divorce of parents, student drinking alcohol, and enhancing classroom environment are beliefs that could enhance student achievement. By so doing, institutions of learning will be in a better position to foster, support, and help students from different states in their education endeavors.

### Limitations and future directions

Besides the favorable outcomes indicated by the proposed method, more is worth mentioning about its shortcomings. One limitation is the use of a particular dataset obtained from Nanjing, China only. The extent to which the results could be generalized to other populations and other contexts of education might also be an issue for future research.

Moreover, the study entailed the use of a number of specified attributes in order to make a forecast as to the academic performance of students. Other variables, for example, extracurricular activities, peer influence, or economic status, could be investigated to get a better understanding of the performance results.

As for future studies, it is possible to consider combining the other machine learning methods or deep learning models to improve the proposed method’s predictive performance. Moreover, exploring the possibilities of real-time tracking and the use of feedback in connection with the model’s outcomes could be also beneficial for educational practitioners.

In conclusion, it can be stated that the proposed method can be considered an effective tool for analyzing student academic achievement and has practical meaning for educational institutions.

## Conclusion

This study utilizes a combination of black hole optimization and Gaussian process regression algorithms to predict students’ academic success in higher education. The method comprises three stages: data pre-processing, identification of effective indicators using BHO algorithms, and application of correlation criteria. Experimental findings demonstrate that this method yields a lower error rate of 0.95 and 0.81 in terms of RMSE and MAE compared to competing methods. Investigations on a dataset including 628 students demonstrated that several factors such as parents’ divorce status, student alcohol consumption, and student behavioral features can impede students’ capacity to achieve academic success. The integration of artificial intelligence technology can aid teachers in analyzing student behavioral patterns, understanding impact mechanisms on academic performance, and developing effective learning supervision plans. Our research indicates that the suggested approach significantly improves the prediction of students’ academic progress. These findings also suggest that artificial intelligence technology might be a useful tool for raising student learning oversight and academic achievement.

## Data Availability

All data generated or analysed during this study are included in this published article.
